# Varied solutions to multicellularity: The biophysical and evolutionary consequences of diverse intercellular bonds

**DOI:** 10.1063/5.0080845

**Published:** 2022-06-01

**Authors:** Thomas C. Day, Pedro Márquez-Zacarías, Pablo Bravo, Aawaz R. Pokhrel, Kathryn A. MacGillivray, William C. Ratcliff, Peter J. Yunker

**Affiliations:** 1 School of Physics, Georgia Institute of Technology, Atlanta, Georgia 30332, USA; 2 School of Biological Sciences, Georgia Institute of Technology, Atlanta, Georgia 30332, USA; 3 Interdisciplinary Graduate Program in Quantitative Biosciences, Georgia Institute of Technology, Atlanta, Georgia 30332, USA

## Abstract

The diversity of multicellular organisms is, in large part, due to the fact that multicellularity has independently evolved many times. Nonetheless, multicellular organisms all share a universal biophysical trait: cells are attached to each other. All mechanisms of cellular attachment belong to one of two broad classes; intercellular bonds are either reformable or they are not. Both classes of multicellular assembly are common in nature, having independently evolved dozens of times. In this review, we detail these varied mechanisms as they exist in multicellular organisms. We also discuss the evolutionary implications of different intercellular attachment mechanisms on nascent multicellular organisms. The type of intercellular bond present during early steps in the transition to multicellularity constrains future evolutionary and biophysical dynamics for the lineage, affecting the origin of multicellular life cycles, cell–cell communication, cellular differentiation, and multicellular morphogenesis. The types of intercellular bonds used by multicellular organisms may thus result in some of the most impactful historical constraints on the evolution of multicellularity.

## I. INTRODUCTION

Multicellular organisms have fundamentally shaped Earth's ecosystems, to the point where we name most biomes by the dominant multicellular organisms living there (e.g., forests, grasslands, and coral reefs). Multicellular organisms contain considerably more total biomass than unicellular life.^1^ Simple multicellular phenotypes appear in our earliest cellular fossils, dating back ∼3.5 × 10^9^ years.^2,3^ Today, multicellular organisms tremendously vary in size and complexity, from just a few cells up to 10^14^ cells per individual,^4^ and from 1 to ∼120 cell types.^5^ In fact, multicellularity has independently evolved many times;^6^ the precise number of known lineages has been increasing over the years as we generate increasingly precise phylogenies of multicellular lineages (see, e.g., Ref. 7). Multicellularity has evolved in all domains of life,^6^ with “complex” multicellularity evolving in animals, plants, fungi, red algae, and brown algae.^8^ The many independent routes to multicellularity demonstrate that there is no “one way” to be multicellular, but rather that this process is contingent on the cell biology of the unicellular ancestor, the details about how nascent multicellular groups form, the environment, and how selection acts on multicellular phenotypes. Because of these independent origins, there is potential to draw general conclusions about multiple routes to multicellularity from a comparative analysis.

While multicellular organisms are exceptionally diverse, they share at least one universal property: they all have mechanisms that keep cells together. As might be expected from their many independent origins, there are many modes of cellular attachment. For instance, cells might remain attached to one another through incomplete cell division processes, or they might adhere with sticky surface proteins, or they might be corralled inside a confining maternal membrane. One way of distinguishing these different cell attachments is by sorting intercellular bonds into two general classes: bonds may be reformable, or they can be permanent (i.e., nonreformable). Extant multicellular organisms sometimes employ a combination of the two classes (e.g., employing permanent bonds at an early stage of life and later shifting to reformable bonds). Conversely, nascent multicellular groups generally form using one of these two bond classes, and their initial intercellular attachment mechanism underpins the starting architecture of the group. The subsequent evolution of multicellular complexity (i.e., form, function, patterning, and differentiation) proceeds in the context of this initial architecture.

Details about the attachment mechanism through which cells form a multicellular group have significant biophysical, ecological, and evolutionary consequences, over both short and long timescales. For instance, on short timescales, bond type impacts the rate at which intercellular bonds form,^9–12^ the topology of connected or physically contacting cells,^13^ and the availability and utility of intercellular space.^14,15^ There are also emergent consequences at the level of the group, for example, how large can the organism grow before intercellular bonds are placed under loads large enough to break them?^16,17^ How does the attachment mechanism impact the geometry of cell arrangements?^18^ How likely are physical forces to fragment entire organisms into separate pieces?^16,19^ How do tissue-level mechanical properties emerge from cellular properties and behavior?^20–25^ How do the type and number of intercellular connections lead to different modes of intercellular communication?^26^ How do nascent multicellular life cycles arise? How do these affect subsequent evolution? The specific class of intercellular attachments leads to different biophysical constraints, advantages, and trade-offs, which we explore in this review.

**FIG. 1.**
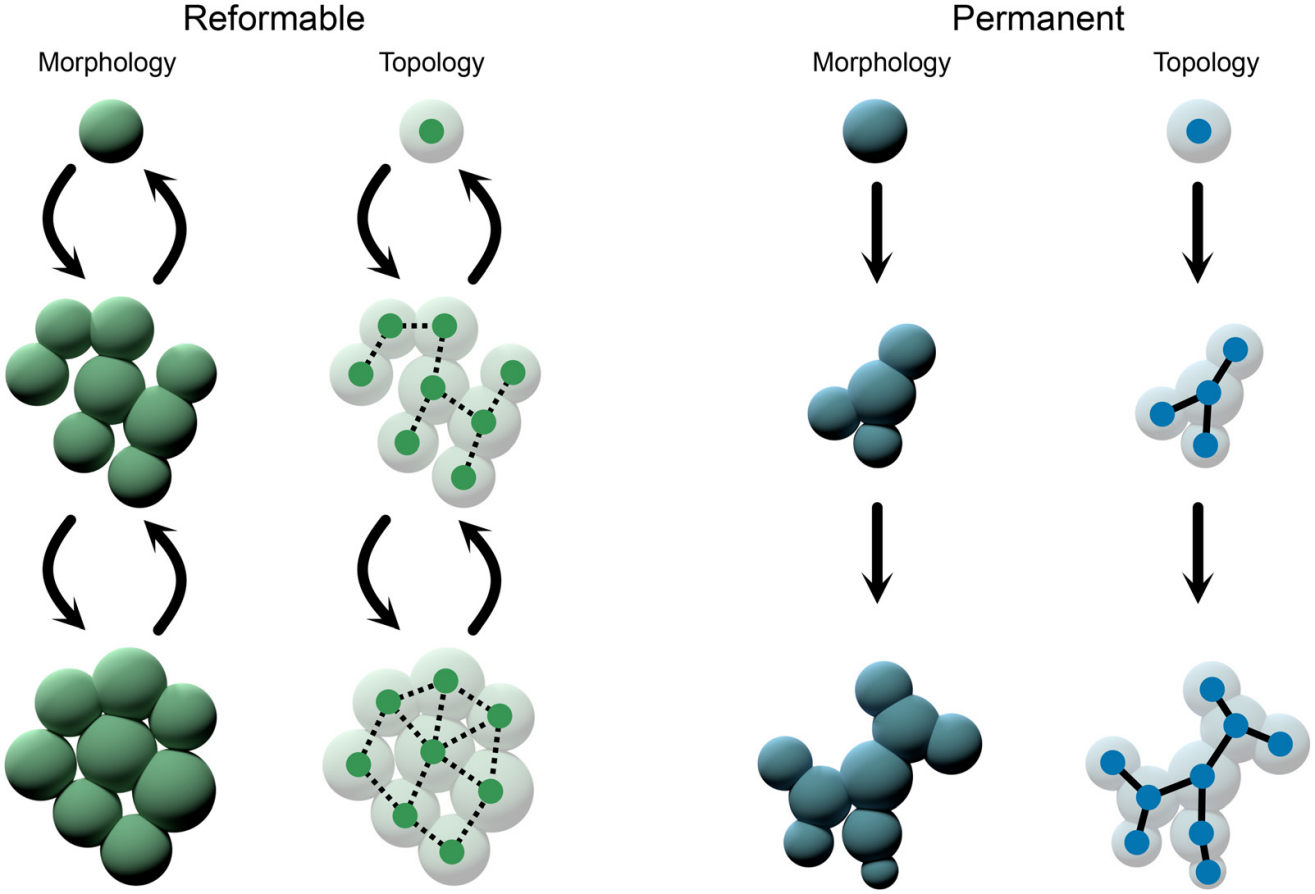
The two main classes of bonds, which form a multicellular organism. Reformable bonds allow for relative cellular rearrangements; permanent bonds do not. This topological constraint has many downstream effects.

In Secs. II–V, we will summarize the two classes of multicellular attachment and discuss their impact on development and evolution (Fig. 1). As different mechanisms have different consequences, we partition the review into four sections: (Sec. II) first, we discuss groups formed with permanent intercellular bonds, how these bonds are formed, their immediate biophysical consequences and constraints such as connection topologies and packing geometries, and some of the downstream effects on communication pathways, (Sec. III) then, we similarly discuss groups formed via reformable bonds, of which extracellular matrix (ECM) and sticky proteins are subexamples, (Sec. IV) we examine the evolutionary consequences of different attachment mechanisms on the evolution of multicellularity, and finally (Sec. V) we discuss some of the ambiguities in the dichotomy introduced here.

## II. PERMANENT INTERCELLULAR BONDS

The first attachment mechanism we will discuss is a “permanent” or “fixed” intercellular bond. This type of intercellular bond is not capable of being reformed if it is broken. Permanent bonds are formed via incomplete cell separation processes. Many multicellular organisms form these bonds through incomplete cell separation. In such processes, mother and daughter cells remain physically attached after the cell division process. This process occurs in both prokaryotes and eukaryotes, spanning many clades of multicellularity: it is observed in bacteria, land plants, green algae, brown algae, red algae, fungi, and in some stages of animal development.^8,27^ It is one of the oldest forms of multicellular assembly^2,28^ and one of the most successful, dominating the planet's biomass.^1^

**FIG. 2.**
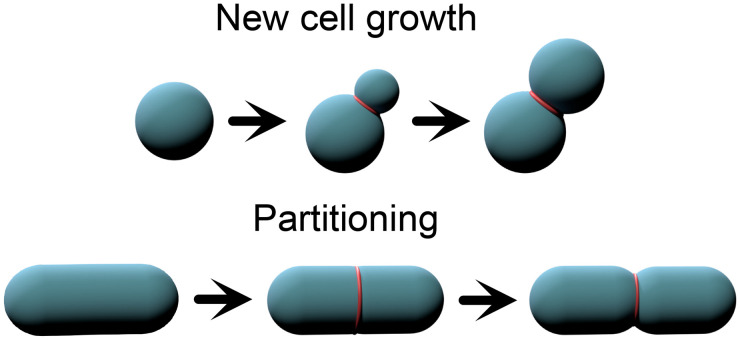
There are two ways to create permanent bonds, both of which involve creating new cells. Either new cells can be grown, and with them, permanent bonds, or previous cells can be partitioned, preserving total volume.

There are a few different versions of permanent bond formation via incomplete cell separation (Fig. 2). Examples include incomplete cytokinesis, where the cell cytoplasms remain connected; incomplete cell separation, where cell cytoplasms may be disconnected, but the cell walls or membranes remain strongly adhered; syncytial growth, where a cylinder of cell wall material is partitioned via crosswalls; and other forms of cell partitioning, where a cell boundary is deposited in the middle of a larger cell, partitioning it into two pieces. In all of these instances, the bonds are formed by cell division, whether that includes additional cell growth or not. Additionally, the bonds cannot be unformed and reformed again; they are fixed until severed, at which point they cease to exist.

### A. Bond Formation (Comparison to Reformable Bond Formation: Secs. III A and III B)

As incomplete cell separation is common across biological domains, details of how it occurs can dramatically differ between organisms. Cell division itself differently occurs in different lineages, the structural components of the cells are different (e.g., plants have cell walls while animals do not), and cell shape and geometry can also vary (e.g., some lineages produce cells that are roughly rectangular prisms, while many others may have spherical or ellipsoidal cells). Additionally, the molecules mediating intercellular attachment are diverse: in most plants, the molecules composing their cell walls include a variety of polysaccharides like pectins and hemicelluloses,^29–31^ while fungal cell walls are composed of different polysaccharides such as glucans, mannans, and chitin;^32,33^ in other cases, protein complexes that span cell cytoplasms provide the structural support of these bonds.^34^

Despite the many differences in the biochemical components of cellular attachment, there are a few important characteristics of the incomplete cell separation process that are broadly shared. For one, the rate of bond formation is intertwined with the rate of cell division, since the division process creates these bonds. Ultimately, this means that these bonds are relatively slowly formed. Second, while not strictly necessary, a common feature of these bonds is the formation of stable cytoplasmic bridges that span from one cell into its neighbor, which can be a key mechanism for intercellular communication. These bridges have been observed in all the different extant taxonomic lineages that exhibit fixed bonds formed from incomplete cell separation. Below, we summarize some cell division processes that lead to the formation of fixed intercellular bonds and highlight the intercellular connections that exemplify the stable nature of these bonds.

#### 1. Land Plants

The vast majority of plant cells develop via incomplete cell division, thus forming intercellular bonds with middle lamella.^30^ At the onset of cell division, a cell plate forms at the center of the dividing cell.^29^ The cell plate grows, thus partitioning the parent cell into two daughter cells. Then, cell wall material is deposited on either side of the cell plate, forming the shared middle lamella. During this process, the cells maintain an intercellular tunnel called the plasmodesmata, which connects neighboring cell cytoplasms.^35,36^ In woody plant tissue, the pectins in the lamellar region become hardened (a process called lignification) to handle the intense tensile and compressive stresses associated with structural forces.^29^ In soft plant tissues, both internal and external compressive forces are generally carried by the cellular turgor pressure, allowing cell walls to be thinner and more flexible; in this case, the cross-linked pectins are generally not lignified.^30^

#### 2. Green Algae

Green algae are a diverse group, consisting of many marine algae and all land plants. Therefore, some multicellular green algae share the same characteristics as the land plants described above. However, green algae can also form intercellular bonds through other processes. For instance, volvocine algae form multicellular groups through a process called multiple fission, where cells first grow to a large size without dividing and then rapidly divide many times, resulting in many cells.^37^ Throughout this process, cells maintain cytoplasmic bridges between their division mates,^38,39^ resulting in an average total of 25 bridges per cell, divided across the several intercellular bonds connecting the cells together.^37^ The bridges are composed of phospholipid bilayers (the same material that composes the plasma membrane), the same that surrounds each somatic cell, and are also characterized by an electron-dense ring.^39,40^

#### 3. Fungi

Many fungi can grow as hyphae, which are characterized by long, branching filamentous structures. Hyphae are the main mode of vegetative growth (i.e., growth that increases the organism's size) for most multicellular fungi. A single hyphal branch is a structural cylinder of the cell wall, mostly composed of mannans and glucans.^33^ When cell division occurs, an internal crosswall called a “septum” grows and partitions the cells within the hyphae. The septum is shared by both neighboring daughter cells. Importantly, cells maintain holes in the septal wall called septal pores.^41,42^ The sizes of septal pores (varying in cross-sectional area from 50 to 500 nm) and the pore density per septum (from single pores to multiple pores) can vary between organisms.^42,43^

Single-celled budding yeast (*Saccharomyces cerevisiae*) readily make the transition to multicellularity under laboratory experimental evolution.^44,45^ In these cases, attached cells share not one cell wall, but rather each cell has its own cell wall, which remains cemented together. For instance, in budding yeast, a chitin-rich region binds the daughter cell to its mother.^32,33^ During the budding process, the two cells share a cytoplasm. In the final stages of cell division, two thick cell walls are constructed between the two cells. In unicellular yeast, the last step in cell division is the dissolution of the chitinous polysaccharide that surrounds the cell junction, thereby separating the two cells, leaving behind a crater-like “bud scar” on the mother cell's surface and a “birth scar” on the daughter cell's surface. However, in cases where proteins, which dissolve the budding chitin scar, are not expressed, the two cell walls remain physically attached and touch one another at the junction.^44,46^

#### 4. Animals and Choanoflagellates

Animals exhibit diverse intercellular bonds that can both spatially and temporally vary. However, stable cytoplasmic bridges are conserved across the embryogenesis process.^47^ In animals from insects to humans, incomplete membrane furrowing during oogenesis and spermatogenesis leaves regions called “ring canals,” which connect their cells.^48–55^ In mammals, these intercellular bridges are typically constructed from midbody matrix protein complexes.^49^ In invertebrates such as nematodes and fruit flies, somatic cells can maintain external intercellular bridges that are formed from actin and other proteins.^47^ External cytoplasmic bridges have also been observed in some protists, like choanoflagellate filaments and choanoflagellate rosettes,^56–58^ which are the most closely related clade of organisms to the animals.^59,60^ Together, it is apparent that incomplete cytokinesis plays an important role in the development of many metazoans and their closest relatives.

#### 5. Multicellular Bacteria

Filamentous multicellular bacteria with permanent intercellular bonds are the first-known multicellular organisms; incomplete cell division began in the cyanobacteria around 2.5 × 10^9^ years ago and was subsequently lost and gained a few times in the ensuing years.^27,61^ Protein complexes span the two cytoplasms of adjacent cells in the bacterial filament.^34^ Similar to the cyanobacteria, *Beggiatoa* grow in filaments that can be found in a variety of marine and freshwater environments.^62^ A different multicellular bacterial family, the streptomycetes, grow in hyphal-like filaments that are then partitioned by septal crosswalls, similar to fungal mycelial networks in the fungi. These crosswalls sometimes have holes through which nutrients and plasmids are transported.^63^

### B. Cellular Spatial Structure (Comparison to Reformable Bond Spatial Structures: Sec. III C)

Microscopic details underlying how incomplete cell division unfolds can produce large macroscopic differences in multicellular topology (i.e., which cells are bonded to which other cells) and geometry (i.e., how much space each cell is afforded). These properties have profound impacts, affecting everything from organismal strength and toughness, to resource sharing, intercellular communication, division of labor, and more.^16,18,26,45^ We call the combination of topological and geometric properties the spatial structure of a multicellular organism.

Since bonds formed via incomplete cell division are, by definition, not reformable, the original bond network cannot rearrange to connect cells that were previously unconnected: organisms are stuck with their original bond network. Furthermore, geometric rearrangements are limited to strictly elastic cases, that is, cases where the cell positions may be stressed into a slightly new conformation, but upon release of the stress, they will spring back to their unstressed state. Intercellular bond topologies can range from filamentous linear networks (e.g., cyanobacteria) to branched networks (e.g., mycelia) and to neighbor networks (e.g., plant meristems). Additionally, there are different types of cell spatial geometries, which can range in dimension (e.g., sheets of cells vs volumes), packing fraction, and more. In this section, we first enumerate different intercellular bond topologies and discuss how they emerge from incomplete cell division; then, we discuss how these bond topologies may affect strength, toughness, and the geometry of cellular arrangement.

#### 1. Topology

##### a. Filaments

One type of intercellular bond topology that can result from incomplete cell division is the linear filament, that is, a chain of cells. In filaments, cells are bonded to a maximum of two other cells. Severing one bond, therefore, results in complete fragmentation of the organism into two distinct pieces, each of which may be viable. This kind of bond topology can result from cell division processes such as binary fission or budding.^64^ Filaments are one of the oldest forms of multicellularity,^65^ including some of the oldest fossils yet found for both prokaryotes^2^ and eukaryotes.^28^ There are also extant forms of filamentous multicellularity, including the prokaryotic cyanobacteria,^27^ and eukaryotic protist choanoflagellates.^56,58^ Formation of filaments, therefore, appears to be a robust and accessible evolutionary strategy.

Filaments present some distinct biophysical constraints. For instance, many organisms have evolved to pass nutrients to their nearest neighbors via cytoplasmic bridges. The bridges then constrain direct resource sharing to only the two nearest neighbors. In cyanobacteria, this constraint led to functional differentiation of cell types that are mutually dependent: cyanobacterial cells can specialize to fix nitrogen or to perform photosynthesis, with cells sharing the products of their activities with their nearest neighbors, leading to a pattern of heterocyst formation.^27^ The linear topology of filaments also has geometric effects: every cell is in contact with the environment, which may include nutrients or toxins. These groups, therefore, do not require additional multicellular structures to channel nutrients from the exterior to interior cells.

In addition to constraints on cell spatial structure and connectivity, filaments present mechanical constraints. The strength of the multicellular structure is equal to the strength of each individual bond; adding more cells adds only one more bond at a time. Therefore, mechanical load (e.g., shear stress) will strain all of the bonds in the filament in series. If any of the bonds fracture, then the entire group splits into two pieces.

##### b. Branched Tree Networks

Another class of intercellular connections is a branched tree network, or branched filament, which is always a planar graph of intercellular connections. Incomplete cell separation can lead to this type of topology when individual cells can maintain connections to multiple daughter cells. Every cell (besides the original root cell) has one basal bond, that is, the bond to its mother; however, cells in these groups can vary in how many daughters they have and remain connected to. Each daughter cell represents the formation of a new “branch.” This type of branched network is common; for instance, it is observed in fungal mycelia,^66^ certain stages of animal development,^67^ *Streptomyces* bacteria,^27^ and also in experimentally evolved “snowflake” yeast.^46^

In branched tree networks, cells can maintain intercellular bonds with more than two other cells, but they are geometrically limited in the maximum number of bonds achievable. If the cells were all equally sized spheres arranged on a 3D lattice with the highest possible packing density, the maximum number of bonds any one cell could have would be 12. In most cases, however, cells are not organized on a perfect lattice but are structurally disordered; this disorder lowers the total number of possible bonds.^68,69^ Conversely, cells generally come in a range of sizes (a property called polydispersity) and they are not incompressible spheres; packing with softer, polydisperse, or nonspherical cells can increase the maximum number of possible bonds.^70^ Geometric cell packing, therefore, plays a clear role in the ultimate topological structure of the multicellular organism.

Similar to linear filaments, branched networks fragment into two separate pieces if any single intercellular bond is severed.^46^ Therefore, increasing the number of cells in the group does not increase the toughness of the organism. In fact, since new cells may physically contact other cells, they can impart mechanical strain, forcing existing cells away from their “relaxed,” that is, unstressed, configuration. Continued cellular reproduction can, therefore, actually decrease toughness, as has been shown in experimentally evolved snowflake yeast.^16^

Branched network topologies may or may not geometrically fill space—in other words, cells occupy a volume fraction $\phi =\frac{Nv_{c}}{V}$ of all space available, where *N* is the number of cells, *v_c_* is the average volume occupied by a single cell, and *V* is the total volume occupied by the organism, including intercellular space. In cases where ϕ<1, also called nonconfluence, there is plenty of intercellular space where nutrients and toxins may diffuse or be transported, potentially accessing every cell of the organism. Mycelial networks, for example, are not generally space filling (see, e.g., Refs. 66 and 71); accordingly, mycelia employ the gaps between their branches to great effect, using this space to dissolve organic matter. Nutrient transport can also be achieved via entirely diffusive processes.^72^ There are also examples of branched tree bond networks that do fill space ( ϕ≈1, called confluence), such as fruit fly egg chambers.^67^ In these confluent cases, an intercellular vasculature capable of transporting nutrients and toxins becomes increasingly necessary as size increases, since diffusion may not efficiently access all cells in the body.

##### c. Neighbor Networks

**FIG. 3.**
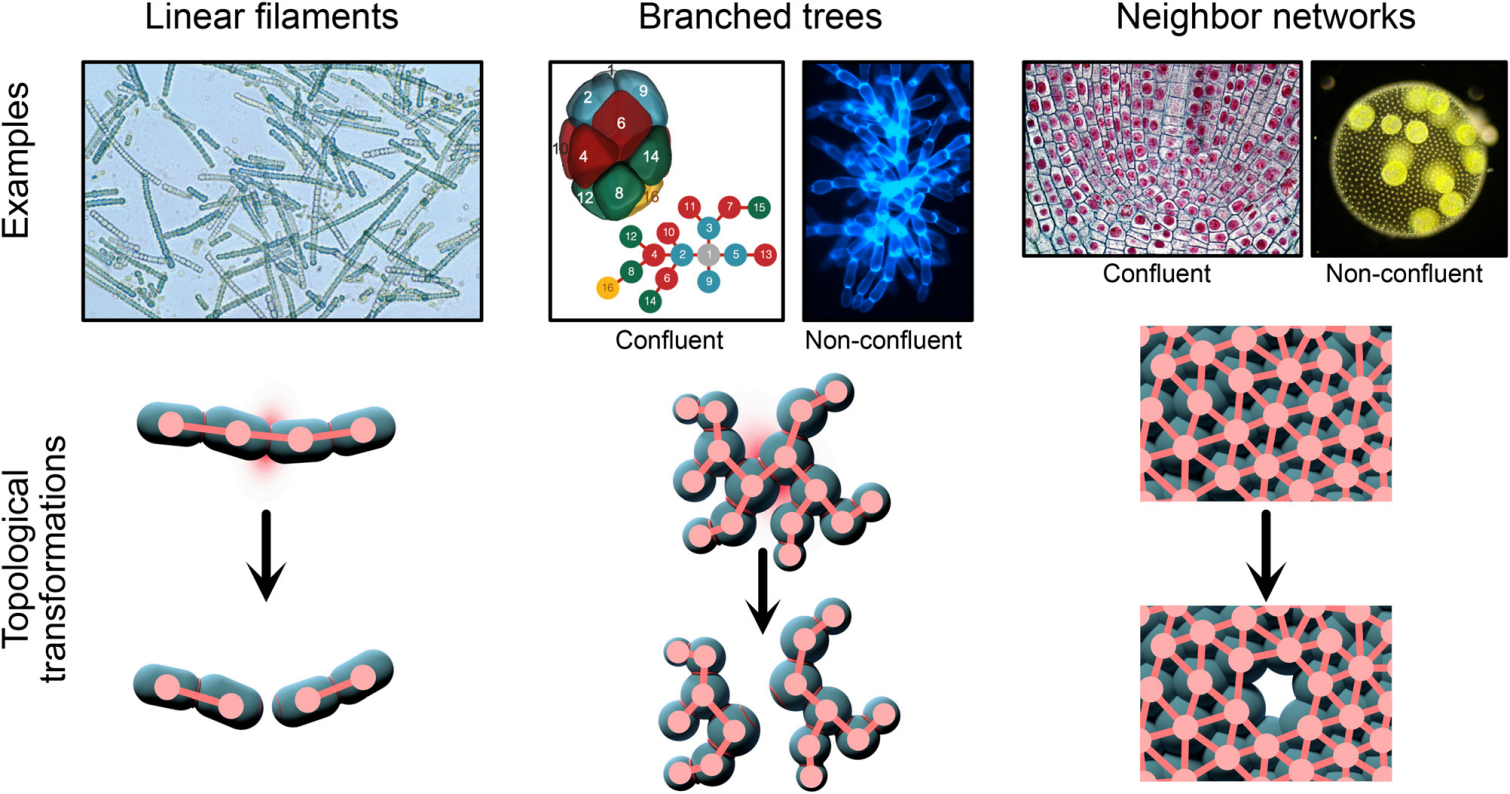
Multicellular groups are formed with linear filament and branched tree bond topologies' fragment into two pieces when any one bond is broken. Neighbor-network topologies do not share this property: multiple bonds must be removed to extract any piece of the organism. Experimental images shown left to right are as follows: (i) linear filaments of the cyanobacteria *Cylindrospermum sp.* courtesy of CSIRO; (ii) membrane-based 3D volume from confocal microscopy of a *Drosophila melanogaster* embryo, courtesy of Dr. Jasmin Alsous, Flatiron Institute; (iii) branching “snowflakes” of the yeast *S. cerevisiae*, adapted from Bozdag *et al.*, bioRxiv: 2021.08.03.454982 (2021). Copyright 2021 Author(s), licensed under a Creative Commons Attribution (CC BY 4.0) License; (iv) the apical meristem in an onion root tip; (v) the entire green algae organism *V. carteri*, adapted from Day *et al.*, eLife **11**, e72707 (2022). Copyright 2022 Author(s), licensed under a Creative Commons Attribution (CC BY 4.0) License.

The third type of intercellular bond topology is a neighbor network topology, in which a cell is connected to its contacting geometric neighbors. As one example, a two-dimensional sheet of cells should be considered, where each cell shares a bond with every neighbor, such as in *Volvox carteri.*^37^ These neighbor networks can, in principle, be either disordered or lattice like. However, the inherent stochasticity of the cell division process, combined with any curvature of the tissue, makes it unlikely that crystalline arrangements of cells will prevail across the entire organism.^18^ In experimental images of select cases, we indeed see that the intercellular bond network has a disorder (see, e.g., Refs. 18 and 73 and Fig. 3). These contact networks can be arranged in two dimensions (like a monolayer cell sheet) or in three dimensions (such as a tube of plant cells).

Contact network topologies can result in confluent tissues (i.e., ϕ=1) or nonconfluent tissues ( ϕ<1). Examples of 2D contact networks that are nonconfluent include some volvocine algae^20^ and possibly choanoflagellate rosettes.^58^ In these cases, there can be significant gaps between the individual cells where nutrients can pass. There are also many examples of confluent tissues in plant tissues. Additionally, it is unclear whether some animal embryos maintain neighbor networks of ring canals or branched tree networks; if they are neighbor networks, then other examples of confluent tissues may be as follows: humans,^52^ rats,^54^ rabbits,^51^ chickens,^55^ frogs,^53^ and fruit flies.^50^ Some animals have somatic intercellular bridges, too: nematodes and fruit flies are the most well studied of these.^47^

When assembled with neighbor networks, organisms do not fragment if a single intercellular bond is fractured. This is because each cell is degenerately attached to multiple others, including cousin cells. In neighbor networks, many bonds must be removed to fragment the organism, meaning that the strength of the tissue is greater than the strength of any one bond. As with branched tree networks, the maximum number of bonds that each cell can achieve depends on the dimensionality of the tissue, shape, and relative size of the cells, and cell compressibility. It may be that not all cells will contact the environment in confluent contact networks, which means that for cells to obtain necessary nutrients, organisms of large size must evolve a vasculature to transport material.^8^

##### d. Special Cases

Some intercellular bond topologies do not neatly fit into one of the above categories. For instance, some fossilized algae, such as those from the rhodophyta^28,74^ or the charophyta,^75^ have cells arranged in clusters of tetrads. Each cell in the tetrad is bonded to two others with a neighbor network topology; tetrads are then bonded one to another in an unknown fashion. It is possible that each tetrad is bonded to the next tetrad at only one location, meaning that the bond topology within tetrads is a contact network, while the bond topology between tetrads may be different (such as a branched tree). That there may be topological networks existing at different modular scales that is an interesting topic for future study.

#### 2. Geometry

Having established that many different intercellular bond topologies are possible for multicellular organisms assembled with permanent bonds, we now turn our attention to how the different arrangements may affect the geometry of cell positions and orientations. This spatial structure inherently depends on the intercellular bond topology. However, any one particular bond topology can be invariant to many different geometric cellular configurations.

Let us consider all the possible configurations of cells in permanently bonded groups. In the extreme case, we might consider a scenario where new cells are randomly positioned, subject to the constraint that they must be bonded according to a prescribed bond topology. Additionally, the cells will be constrained by their geometric size: two cells cannot occupy the same space. Nonetheless, there are very many (in fact, uncountably many) different ways that the cells can be positioned subject to these constraints. If every configuration is equally likely, then saying anything quantitative about the cell structure may seem intractable; however, the maximum entropy principle can provide precise predictions of quantities such as the space afforded to each cell. In the experimental studies of multicellular organisms, (i) snowflake yeast, which form groups with branched tree topological structures, and (ii) *V. carteri*, which form groups with a cousin network topological structure (see Fig. 3), the observed distribution of volume per cell was found to match the predicted distribution from maximum entropy considerations.^18^ Their cellular spatial structure was remarkably reproducible, even without explicit developmental patterning, underpinning the emergence of novel, heritable multicellular traits that arise from the mechanics of cellular packing.^18,76^ Entropic effects on cell packing are not only simply a factor for small, undifferentiated groups of cells but have also been observed to affect organisms that possess complex developmental regulation. For example, cell packings in fruit fly embryos are known to follow patterns that arise from the entropy of “frustrated” topological configurations.^67^ Deviations from maximum entropy predictions, whether these are geometric or topological in nature, also provide important information about the underlying processes leading to multicellular assembly. For instance, deviations can indicate where developmental patterning is strongly affecting morphology.^18^ Maximum entropy predictions may, therefore, become a tool for investigating the origin and extent of developmental regulation.

### C. Bond Fracture (Comparison to Reformable Bond Breaking: Sec. III D)

By definition, fixed intercellular bonds are not reformable. As a consequence, cells with these types of intercellular bonds are not motile with respect to one another: they cannot rearrange their topological connections, significantly limiting their ability to spatially move. Any forced rearrangement event causes permanent structural damage. In permanently bonded organisms, mitigating or controlling the frequency of bond fracture is an essential part of achieving structural robustness.

The forces that cause bond fracture can come from internal or external sources, and can emerge and propagate over a wide range of magnitudes and length scales inaccessible to single cells. For instance, external shear forces can arise from fluid flows and wind loads; neighboring multicellular organisms may apply forces on one another; in some cases, predators can apply forces on their prey (or vice versa); for large multicellular organisms, gravitational forces become relevant. These external forces can fragment multicellular organisms.^58,62,77–79^ Furthermore, internal stress from cell division can lead to large, heterogeneous intercellular force networks due to cell crowding^80^ and can eventually lead to fragmentation.^16^ For example, in experiments on confined single-celled yeast, large and heterogeneous forces arose from continued cell division within confinement.^17^ The boundary conditions imposed by the walls resulted in a self-driven jammed cellular configuration; without the confining walls, the single cells would have rearranged into a configuration with less internal stress. Multicellular organisms assembled with fixed bonds do not require confinement to achieve the same high-stress effect: the bonds prevent rearrangements, allowing stresses to persist and grow until bonds fracture. To control bond fracture, multicellular organisms must confront both external and internal kinds of physical stresses.

There are four basic strategies that can control the frequency of fracture due to either internally or externally generated forces. First, some organisms have evolved mechanisms, which can correct and mend broken intercellular bonds, but the mended bonds are not formed through incomplete cell division.^81^ Furthermore, these new bonds may be formed using different adhesion molecules than the initial bond. Since it presumably takes time to evolve additional cellular adhesion mechanisms, it is possible that nascent multicellular organisms formed via incomplete cell division may not possess corrective mechanisms for intercellular bond fracture.

In a second strategy, organisms may change the toughness of their intercellular bonds. For instance, in woody plant tissue, intercellular bonds have evolved to become strong and tough through lignification processes that can weather large shear and compressive stresses necessary for tall organisms, like trees, which experience gravity, wind load, and more.^30^

The third method of mitigating bond fracture is by modifying the number of intercellular bonds. Partially, this ability is encoded in the different types of intercellular connection topologies. For instance, linear filaments with *N* cells are formed with *N*–1 intercellular bonds. By contrast, a bond network arranged on a cubic lattice will have six connections for each cell, therefore resulting in a higher bond-to-cell ratio. Organisms may also increase the number density of their bonds by producing, for example, multiple cytoplasmic bridges connecting cells rather than just one (as one example, up to 25 bridges connect neighboring cells in volvocine algae^37,39^).

Finally, multicellular lineages may modulate cell-packing density. For instance, in laboratory-evolved strains of snowflake yeast,^44^ continued selection for large size led to morphological changes in cell shape.^16^ Cellular elongation resulted in a reduction of the packing fraction in these groups and, therefore, reduced cell crowding and mitigated stress accumulation. When daily selection for larger group size was extended to 600 days (3000 generations), the cell shape mutations became a dominant feature of the organisms, leading to highly elongated cells^25,45^ that persisted even under diverse growth conditions. Changing cell-packing fraction can, therefore, be a highly effective strategy for controlling bond fracture rate, in some cases outperforming the strategy of simply strengthening intercellular bonds.^82^

### D. Fragmentation as Multicellular Reproduction

Reproduction is a necessary component of Darwinian evolution; for multicellular groups to become Darwinian individuals, they must be capable of creating offspring. While many multicellular organisms reproduce via complex processes involving many levels of genetic, biochemical, bioelectric, and mechanical signaling,^83–86^ many others reproduce via fragmentation. Fragmentation into viable propagules, each with stable intercellular bonds formed by incomplete cell division, is a common form of asexual multicellular reproduction in plants^78,79^ and bacteria,^62,77^ and has been observed in the fungi^44^ and choanoflagellates.^58^ Given the mechanistic simplicity and phylogenetic dispersion of this strategy, it is possible that fragmentation is one of the earliest strategies of asexual multicellular reproduction.

As noted above, fragmentation can be externally^79^ or internally driven.^16^ For groups with permanent bonds, especially branched trees, fragmentation is a simple mechanism of multicellular propagation that can arise as an emergent property of cellular growth within the geometric constraints of a multicellular cluster. In linear filaments and branched trees, fracturing a single bond leads to complete organism fragmentation into two separately viable propagules, each of which encodes the genetic information of their unfragmented parent. Therefore, in some cases the process of fragmentation is a simple, easily evolved mechanism underlying group reproduction and the origin of early multicellular life cycles (see, e.g., Ratcliff *et al.*^46^).

### E. Intercellular Channels (Comparison to Reformable-Bonded Intercellular Communication: Sec. III F)

One of the benefits of multicellularity is the ability for cells to communicate and divide labor, exchanging nutrients, chemical signals, or even entire organelles from one cell to another. While these interactions can be entirely external to the cells (e.g., they may excrete a chemical signal to diffuse to all neighboring cells, a la quorum sensing), it can be beneficial to have a targeted interaction pathway that connects two or more cells, allowing them to privately exchange goods. Doing so protects otherwise common goods, reducing the potential for social conflict. As we describe above, targeted intercellular channels such as cytoplasmic bridges are a feature of many multicellular organisms that evolved to form fixed cellular bonds, including many of the most diverse and complex multicellular lineages.^8,35,36,47,87–89^ Cells connected via bonds from incomplete cytokinesis already have a built-in pipeline for targeted cell–cell communication. The relative ease of forming these communication channels may have been an important step in the evolution of multicellularity. Recent studies have demonstrated that the types of intercellular communication networks formed by permanent bonds may be particularly advantageous for evolving a reproductive division of labor.^26^ Thus, these communication channels are not only easy to form, but also they facilitate differentiation in ways that fully connected networks (like those from public resource sharing) cannot.

## III. REFORMABLE BONDS

**FIG. 4.**
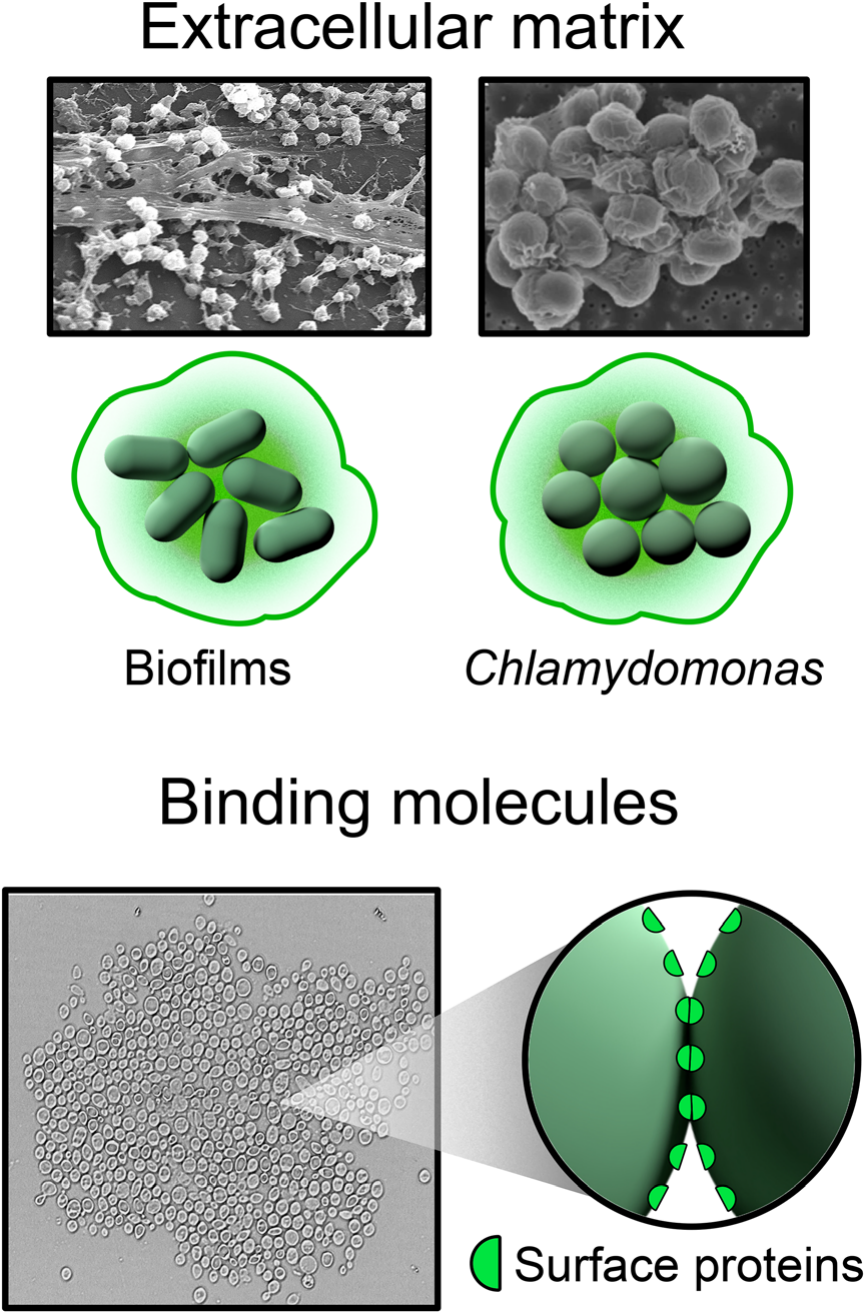
There are two main types of reformable bonds. One type is a sticky extracellular matrix that surrounds the cells. A second type is a surface-binding protein that can interact with proteins on the surface of another cell. The upper two experimental images are (i) an electron micrograph of a biofilm of *Staphylococcus aureus* bacteria on a catheter and (ii) a scanning electron micrograph of a multicellular colony of *C. reinhardtii*, adapted from Herron *et al.*, Sci. Rep. **9**, 2328 (2019). Copyright 2019 Author(s), licensed under a Creative Commons Attribution (CC BY 4.0) License. The bottom experimental image is an aggregate of flocculating yeast, *S. cerevisiae*.

Aggregational adhesion is the process of initially attaching separate cells together with reformable bonds (Fig. 4). There are two broad classes of reformable bonds: cells may excrete an extracellular matrix (ECM), which surrounds them and binds them together like a viscoelastic “glue,” or cells may express sticky, velcro-like surface proteins that interact with proteins or other molecules on the surfaces of other cells. Both mechanisms are extremely common in nature. They are also often both simultaneously present or present along with permanent bonds (e.g., rosette-forming choanoflagellates^58^) As these bonds readily re-form after breaking, cells can rearrange, actuating a dynamic multicellular structure with rich physics and biology. In this section, we briefly review ECM and sticky protein formation mechanisms, and then discuss the emergent physical and biological properties that arise from dynamic rearrangements. We also indicate the profound implications reformable bonds have on the subsequent evolution of multicellular lineages.

### A. Extracellular Matrix (ECM) (Comparison to Nonreformable Bond Formation: Sec. II A)

The extracellular matrix is a broad family of secreted proteins, polymers, and polysaccharides, which act as a “mortar” that provides biophysical and biochemical scaffolding for the cellular “bricks” embedded within it. It is prevalent in a broad range of multicellular collectives, including bacteria, fungi, animals, algae, and plants. ECM thus refers to a wide range of different materials, with highly varied compositions. Individual organisms can even express highly heterogeneous ECM, with a composition that spatiotemporally varies as it engages cells in continuous biochemical and biomechanical interactions.

Of the many different types of organisms that employ ECM to attach cells in a group, we focus on two archetypal examples: bacterial biofilms and animal tissues.

#### 1. ECM Composition

The extracellular matrix is comprised of multiple, diverse interacting biomolecules,^90,91^ including polysaccharides, proteins, dead cells, lipids, and extracellular DNA.^92,93^ In bacterial biofilms, polysaccharides compose the majority of ECM mass;^94^ these polymeric chains form a network that binds cells to the surface and to each other.^95^ In animals, proteoglycans and fibrous proteins such as collagens, elastins, and fibronectins form the majority of the matrix.^90,96,97^ Nonenzymatic proteins may allow cell surfaces to bind to the polysaccharide network,^98,99^ while their enzymatic counterparts may then degrade these biopolymers for consumption by the cells in the case of starvation.^100^ In addition, the debris of dead cells may remain stuck within the extracellular matrix long after cells die.^101^ Extracellular DNA also forms an important component of the ECM; it has been implicated in both structural and evolutionary processes involving bacterial biofilms and their resistance to removal in infections.^102–104^ While the formation of an extracellular matrix has been observed in different species of bacteria and even in polymicrobial communities, the composition and structure vary between single and multispecies colonies.^105^

No matter the exact composition of the ECM, it provides essential and rich physical and mechanical properties to the group,^106,107^ and provides protection to the individual cells it encases. In Secs. III A 2–III A 4, we will describe some different kinds of ECM-attached multicellular groups and explore the different properties that the matrix provides the system, and its evolutionary consequences.

#### 2. Biofilms

Biofilms are surface-attached communities of bacteria, fungi, and/or archaea^108^ held together by an ECM.^91^ Biofilm formation starts when cells irreversibly attach to a surface.^109–111^ As the cells reproduce, they secrete polysaccharides and other biomolecules that strengthen their attachment to both the surface and each other. This process forms highly heterogeneous three-dimensional structures.

Biofilms can confer distinct benefits and disadvantages to microbes living within them compared to their planktonic counterparts. For instance, microbes living in a biofilm have a slower growth rate due to oxygen and nutrient limitations; however, individual cells in biofilms are also less susceptible to fluctuations in environmental conditions. The ECM enclosing the biofilm provides a protective microenvironment, shielding microbes from, for example, phages and antibiotics.^112,113^ It also provides mechanical protection in situations where the biofilm is exposed to shear stresses or mechanical pressure.^91,114,115^

Biofilm formation also enables many complex behaviors that are analogous to multicellular processes. For instance, some biofilm colonies spatiotemporally partition cell behavior such as programmed cell death^27^ (where some cells are cannibalized to enable the remaining cells to access their nutrients), division of labor,^116^ and sporulation (where cells that are part of distinct spatial structures are more likely to sporulate). In other cases, biofilms can construct well-defined channels that may facilitate the transport of liquid nutrients and waste over large distances.^117,118^

#### 3. Animals

In addition to the diverse range of biofilm communities that use the extracellular matrix to hold collectives of cells together, animals often employ ECM as an attachment substrate for their epithelium. Generally, underneath the epithelial cells is a dense collagen-rich matrix called the basal lamina. This layer employs a variety of proteins, such as integrins, fibronectins, and elastins, to link cells to the ECM and thus to the rest of the collective.^90^ The genes that encode for these proteins are found in all major animal phyla including sponges,^119–122^ suggesting that the first multicellular animals may have been formed through ECM adhesion;^59^ at the least, the last common ancestor of extant animals likely had the capability of reformable cell–cell adhesion through ECM. Consistent with this view, some of the closest relatives to animals, the choanoflagellates, not only employ permanent intercellular bridges to adhere cells one to another but also use a spherical core of ECM as an important biophysical structure, which cements a “rosette” of cells together.^56,58^

#### 4. Experimental Evolution of Multicellularity via ECM

One of the best-studied clades of multicellular organisms is the volvocine green algae. ECM production underlies the formation of spherical multicellular structures in the most sophisticated volvocine green algae.^123^ Unlike most clades of multicellular organisms, the volvocine green algae contain species that exhibit the full range of multicellular complexity, from the single-celled *Chlamydomonas reinhardtii* up to the macroscopic *V. carterii*, which displays genetically regulated germ–soma differentiation.^37^ Experimental evolution of *C. reinhardtii*, either via coculture with a gape-limited predator^124^ or selection for rapid sedimentation,^125,126^ readily forms simple multicellular groups in which cells are attached via a secreted ECM. In some lineages of these experiments, newly multicellular groups formed an alternating unicellular/multicellular life cycle, where single cells detach from the group, disperse, and then grow new multicellular groups.^124,125^ In other lineages, multicellular clusters propagate by fragmenting into multiple multicellular clusters.^124^ The rapid evolution of these algal groups in the laboratory demonstrates that reformable cellular bonds, such as those mediated by an ECM, can be a first step in the transition to multicellularity.

### B. Sticky Surface Proteins (Comparison to Nonreformable Bond Formation: Sec. II A)

In many multicellular organisms, intercellular adhesion is mediated by a battery of sticky, reformable proteins that attach one cell surface to another. This method of intercellular adhesion is fundamentally different from the secretion of an extracellular matrix. As these proteins only exist on the cell surface, cells must be directly in contact for these proteins to interact and bind them together. In some cases, this process spurs the two separate cell surfaces to weld together in tight formation via adherens or tight junctions.^127^

While incomplete cell division always results in groups with high relatedness, aggregation via sticky proteins can result in genetically diverse groups.^9^ In the absence of either a highly structured local population^128^ or a mechanism of kin recognition (see, e.g., Ref. 129) aggregative groups will often be composed of cells that are no more related to each other than would be expected by chance, limiting the potential for selection to act on group-level traits.^130^

There are many types of sticky surface protein bonds that occur across the domains of life. For example, sticky surface protein aggregation is observed in bacteria,^131^ fungi,^132^ slime molds,^133,134^ and animals.^8^ Each of these cases differs in its composition, strength, and selectivity. Nonetheless, they share at least one common property: sticky surface protein bonds can drive rapid group formation. Facultative multicellular life cycles, in which group formation occurs in response to an environmental stimulus (e.g., starvation), thus often utilize rapid, reformable cell–cell bonds.^131,134,135^

#### 1. Examples of Sticky Surface Protein Aggregation

##### a. Yeast

The aggregation of yeast cells, known as “flocculation,” has been well studied in *S. cerevisiae* in part because flocculation enables yeast to be removed from beer and wine after fermentation is performed.^136^ Flocculation in yeast is caused by several structurally similar genes, including *FLO1*, *FLO5*, *FLO9*, and *FLO10*, with *FLO1* receiving the most attention.^137^ Once activated by Ca^2+^ ions,^138^ these proteins form a reversible cell–cell bond by binding to the mannose sugars present on the surface of another yeast cell, regardless of whether that cell is expressing flocculation proteins.^139^ Flocculation is additionally sensitive to environmental conditions such as temperature, pH, and nutrient availability.^136^ The apparent redundancy of the FLO genes enables variable control over the flocculation phenotype; each of the proteins has the ability to bind different sugars.^140^ *FLO* proteins enable *S. cerevisiae* to coflocculate with non-*Saccharomyces* species; expression of different *FLO* proteins produces varying degrees of specificity in flocculation phenotype.^141^ In contrast to the sugar-binding FLO genes, FLO11 proteins bind each other, allowing for the potential to use this not just as a mechanism of cell–cell attachment but also kin recognition, as more FLO11 from more closely related yeast strains have a higher binding affinity.^142,143^

##### b. Animals

One of the characteristic features of animals is the epithelial tissue that surrounds their multicellular bodies. The cells comprising this tissue generally adhere to one another through sticky surface proteins. The archetypal example of sticky surface proteins in animals is cadherins, which generally bind neighboring epithelial cells one to another; integrins then bind the entire epithelial layer to the basal lamina that resides beneath the surface.^59,144,145^ Cadherins of one cell can interact with cadherins on neighboring cells to form an adherens junction.^146^ Alternatively, they may directly and indirectly bind with catenins, cytoplasmic proteins that stick out from the cell surface.^147^ These bonds rapidly form and quickly strengthen: cells adhere within seconds of first contact, and the force required to separate the cells increases fivefold within ten minutes.^148^ In some cases, the adherens junction leads to the formation of a stronger bond, called the tight junction, that uses an entirely different set of cell-surface proteins such as occludins, claudins, and ZO proteins.^147^ Other surface interactions that animals utilize to adhere to cells include desmosomes, which connect cytoskeletal filaments that extrude from the surface of two cells.^127^

### C. Cellular Attachment Geometry and Topology (Comparison to Nonreformable Bond Spatial Structures: Sec. II B)

No matter the specific binding interactions, whether through ECM production or via sticky proteins, bonds in aggregative groups are reformable, so cells can rearrange their positions. As a result, the nearest neighbors of a particular cell will be time dependent. This is a fundamentally different situation than multicellular groups assembled from incomplete cell separation processes, since neighboring cells in those groups are “frozen” in place. Rearrangements in cell position, therefore, lead to fundamentally different biophysical constraints.

In groups formed with, for example, sticky surface proteins that require cell–cell contact, what sets the number of contacts per cell? Each cell has a minimum and a maximum number of contacting cells, set by cell-packing constraints. To be part of a group, each cell must be attached to a minimum of at least one other cell. The maximum number of contacts is geometrically limited; for instance, in 3D space with monodisperse (i.e., same size) spheres, the maximum mean number of contacts is 12, corresponding to maximum density lattice packing. Yet, multicellular assemblies are generally disordered, which dramatically lowers the mean coordination number.^68,69^ Conversely, cells are generally neither incompressible, nor monodisperse, nor spherical; packing with softer, polydisperse or nonspherical cells can increase the number of contacts.^70^

The ECM is, in general, less restrictive than sticky surface proteins; while sticky proteins require cell–cell contact, ECM acts as a glue between cells, meaning direct physical contact is not strictly required. Nonetheless, the maximum coordination number is still subject to the same geometric cell-packing constraints. In fact, cell density is experimentally observed to steadily increase over the lifespan of a biofilm, partially due to compression stemming from surface tension-like forces.^14,149^ Cell packing thus becomes an important physical limit for aggregative groups, constraining their density and the number of intercellular contacts. Such geometric constraints can then proceed to underlie the complex structural properties of biofilms. For example, bacteria have many mechanisms with which they kill each other, such as the harpoon-like type VI secretion system.^150^ Geometric cell packing constrains the number of cells in direct contact, which, in turn, limits the efficacy of contact-killing mechanisms.^151^ Furthermore, the accumulation of dead cells and dead cell debris further inhibits intercellular contact, preventing or dramatically slowing the killing rate.^101^ When stagnant biofilms are mechanically mixed, killing resumes, demonstrating that the contact network provides important ecological structure to aggregative communities of cells.^101^

**FIG. 5.**
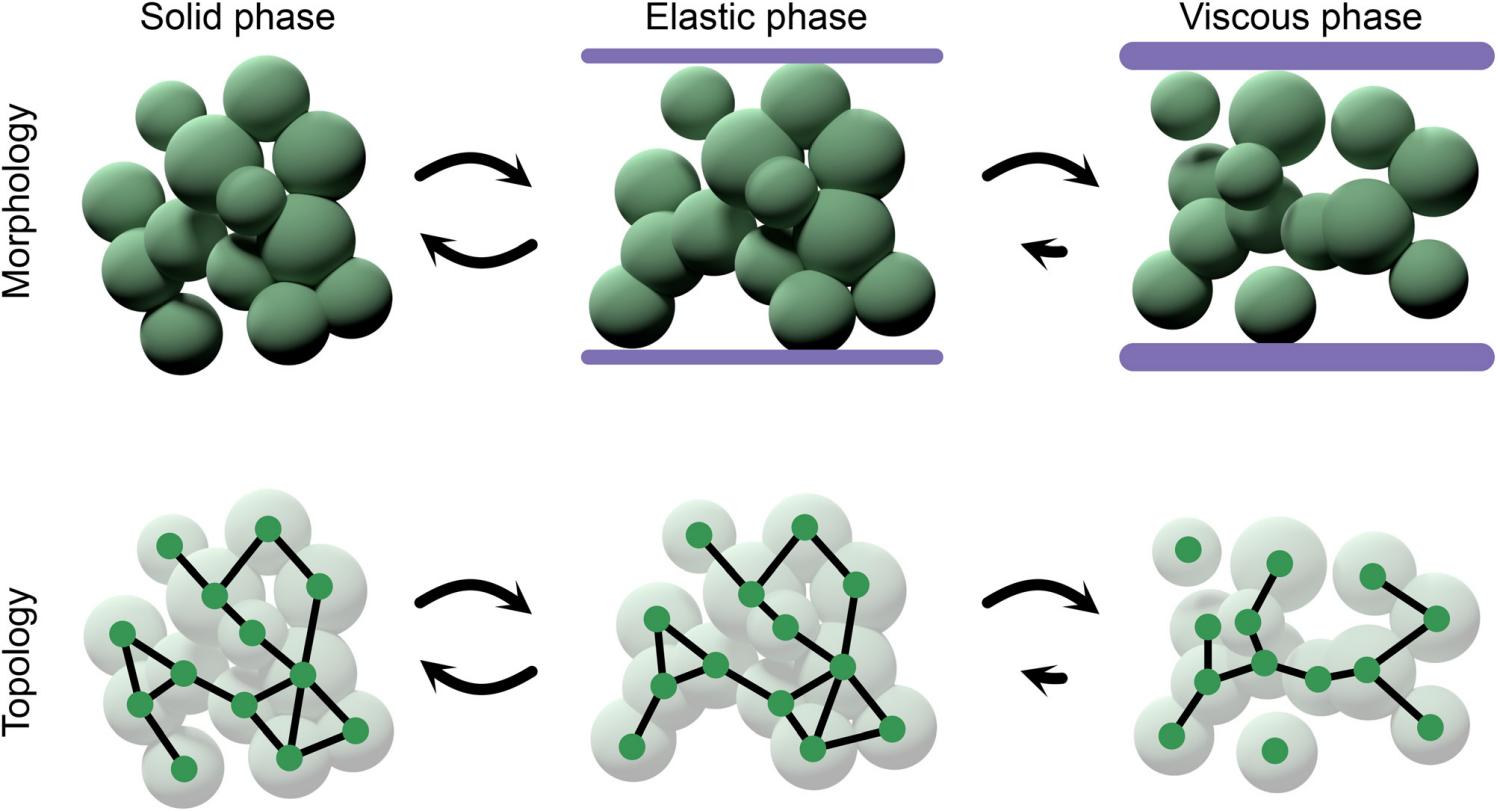
Reformable bonds lead to a rich diversity of cellular arrangements, even within one organism. Under small strain, bonds will not rearrange; under middling strain, bonds may plastically rearrange; and if the strain is large enough, it may cause many rearrangements of the network topology.

In addition to the physical constraints of cell packing outlined above, dense cell packs can inhibit the diffusion of nutrients and toxins^72^ and mechanically impact the cell cycles of cells embedded in the dense pack.^17^ Furthermore, as discussed in more detail below, the mechanical properties of groups with reformable bonds are dynamic and diverse (Fig. 5).

#### 1. Cellular Spatial Structure

In nonconfluent (i.e., cellular packing fraction, ϕ<1) ECM-mediated aggregates, such as in bacterial biofilms or flocculating cells, the packing fraction steadily increases as cells reproduce.^149,152^ Nonconfluent, sticky protein-attached aggregates exhibit similar behavior with one important caveat; since ECM does not require direct cell–cell contact for adhesion, sticky protein aggregation can only occur at a higher packing fraction than in the ECM groups. Nonetheless, both types of aggregates exhibit an increase in packing fraction as cells reproduce. As cellular-packing fraction increases, spatial structure is increasingly dominated by contact biomechanics arising from cellular growth and reproduction. Eventually, aggregates can exhibit jammed packings, reminiscent of granular materials.^17^

At high packing fractions, nonspherical cells (e.g., rod-like bacteria) tend to align.^152^ Nematic and smectic orderings, or the alignment of particles within a suspension, have been observed in many physical systems,^153,154^ including nonequilibrium, active matter.^155,156^ In equilibrium systems, these phases of particle alignment are entropic in nature;^154^ that is, of all possible configurations of rod-like particles (positions and orientations), most of the allowable configurations are aligned.^157^ In living, active systems, it may not be fair to claim that these effects are entropic in nature, since the activity of cell division causes rearrangements and fluidization. Nonetheless, living cellular aggregates that exist at relatively high packing fractions are prone to alignment, even as cell growth and death push the system out of equilibrium. When confined to two dimensions,^152^ these active nematic aggregates produce topological defects,^23^ an important hallmark of both passive and active liquid crystals.^154,155^ In three dimensions, orientational ordering proceeds in a stereotyped manner, with surface-level cells achieving high nematic ordering parameters early in biofilm development and later causing cascades of cell alignment.^14,24,149,158^

In addition to orientational ordering via depletion forces, maximum entropy considerations can accurately and precisely predict the distribution of volume per cell, particularly as contact forces become more important than other interaction forces. The reason for this is that at the jamming point (i.e., when hard, athermal grains become locked in place via contacts with their neighbors), hard-sphere configurations become equiprobable.^159^ Subsequently, precise predictions can be derived for the amount of space associated with each cell in the pack.^160^ The predicted distribution has been experimentally validated for hard grains, foams, and organisms formed with fixed intercellular bonds;^18,160,161^ in simulations, Day *et al.*^18^ also confirmed that cellular groups with sticky interactions may pack cells according to the maximum entropy distribution, too. Experimental work confirming this packing distribution in biofilms or sticky protein aggregates remains open for exploration.

In a related vein, recent studies quantified the spatial structure of aggregates by analyzing nearest-neighbor topology.^162,163^ These works use the “topological Earth mover's distance” to compare local graph neighborhoods of cell centers, finding that, for example, different biofilms can be strictly distinguished based upon static topological information.^163^ They have also extended this capability to extract the steps in a dynamic developmental process concerning aggregative epithelial tissue.^162^ Future work may combine these topological approaches with the geometry of the spatial structure to provide considerable insight into the dynamics and formations of aggregative cell groups.

### D. Frequency of Rearrangements and Adhesion Strength (Comparison to Nonreformable Bond Fracture: Sec. II C)

In principle, reformable bonds can break, allowing cells to rearrange and then re-form, connecting new pairs of cells. In practice, however, the frequency of cellular rearrangements varies and is context dependent. For example, animal tissues are known to alternate between states of fluidity and rigidity during development;^22,164^ the onset of rigidity is associated with the loss of the ability to rearrange neighbors. Studies of epithelial tissues, such as human bronchial cells and drosophila ventral cells, show that mature, uninjured, and nonmalignant epithelial layers generally approach a disordered and jammed solid state.^22,165,166^ Nonetheless, cell rearrangements are commonly observed in many tissues during development in animal embryos,^164,167^ wound healing,^168^ injured or cancerous growths,^169^ and even in mature tissue layers grown *in vitro*.^170^

One reason why cellular rearrangements are common is that rigidity is readily destroyed by cellular activity. For example, any amount of cell division or cell death fluidizes tissues,^171^ from epithelial layers^172^ to bacterial biofilms.^173^ Cellular motility can also drive rearrangements.^169,170^ Unlike birth–death activity, a critical threshold of motility must be reached for fluid-like rearrangements to be achieved.^174,175^ Fluidity driven by self-propulsion in tissue layers with multiple cell types has also been observed to result in slightly demixed configurations, where cells are more likely to be located near the cells of the same type.^176^ The dynamics associated with rearranging tissue monolayers are commonly studied through vertex models, where different classes of cell rearrangements are labeled as, for example, T1 or T2 transitions,^172,176,177^ reviewed in Ref. 178.

Cellular rearrangements in these tissues can occur at a high frequency as the adhesion strength of reformable bonds in animal tissues is generally fairly low (though strength varies depending on the organism, cell type, and environmental factors). However, the literature is sparse on this topic; a small number of studies have been published on the mechanical measurements of animal cell adhesion, with even fewer published on plant cell adhesion. Nonetheless, it is worth comparing the order of magnitude estimates from animal and plant studies of cell separation forces. A literature review of several separate studies estimates that the force per unit area required to separate two adherent animal cells is on the order of 10–1000 Pa.^148,179–183^ The wide range of values is likely due to the fact that these measurements were made with cell types including kidney cells, human red blood cells, human white blood cells, zebrafish endoderm cells, and mouse sarcoma cells. We can compare these measurements to measurements of the force per area required to fracture onion tissue. Onion tissues are held together with nonreformable middle lamella. The force per unit area required to fracture the onion tissue was found to be 335 MPa,^184^ about 10^5^ times greater than the strength of reformable animal bonds. In experiments of grafted benth (*Nicotinia benthamiana*, a close relative of tobacco) cells (i.e., plant cells held together with reformable bonds), researchers measured the separation stress to be about 20 KPa, about 10^4^ times weaker than the nonreformable onion bonds.^185^ In addition, experiments with baker's yeast imply that yeast groups formed with chitinous, nonreformable bonds are stronger than yeast groups formed with flocculation proteins that adhere to cell surfaces. For instance, vortex mixing of chitin-bonded clusters does not destroy the cluster,^44^ while vortex mixing is known to destroy flocculated groups.^12^ While not conclusive, these observations support the idea that reformable bonds are generally weaker than nonreformable bonds.

### E. Mechanical Properties of Rearranging Cell Networks

Cellular rearrangements have mechanical and material effects, which often hold biological consequences. In conventional materials such as fluids or solids, the mechanical properties of the material are heavily dependent on the type, number, and strength of interactions between particles. The same is true for active cell networks, including biofilms and sticky aggregates.^24^ Intercellular interactions facilitate continuum descriptions of multicellular mechanical properties such as tissue fluidization,^171^ height fluctuations,^173,186^ the onset of rigidity,^80^ elasticity,^187^ and wrinkling.^188^

Ultimately, the electrostatic interactions caused by either sticky surface proteins or ECM lead to complex, viscoelastic behavior, that is, they can viscously and elastically respond.^167,189^ Furthermore, unlike nonliving glasses, foams, or gels, these living aggregates are active, as cells reproduce, die, and move. The active viscoelasticity of tissues and aggregates has been the focus of a broad research thrust.^106^

#### 1. Elastic-Like Properties

Even when assembled with reformable bonds, multicellular tissues and aggregates are fundamentally elastic on some time and energy scales. For instance, when exposed to small external stresses, tissues elastically rebound.^167^ Aggregated colonies can exhibit hallmarks of elastic solids, such as wrinkling and buckling,^188^ which can be driven by cell death and reproduction.^190^ Wrinkling and buckling have been implicated as an important step in many developmental processes, including furrowing and folding in complex multicellular organs.^191,192^ Tissues are often modeled as elastic solids on some timescales^187^ due to these properties.

#### 2. Viscous-Like Properties

Unlike multicellular groups assembled with nonreformable bonds, reformably bonded groups can display viscous properties due to their ability to rearrange. For example, externally applied forces can cause shear flows, where neighboring cells slide past one another and make new interactions with new cell neighbors.^80,167,193^ Multicellular aggregates can also exhibit an effective surface tension.^21^ Furthermore, differential adhesion between different cell types can cause cells to phase-separate like oil and water droplets.^21,194–197^ Such fluid-like properties, when combined with the elastic properties that exist on shorter timescales or smaller energy scales, yield an active viscoelastic solid.

#### 3. Glass-Like Properties

Last, growing cell layers, especially in confinement, have been shown to resemble glass-like dynamics in a number of different contexts. For example, tissues can “freeze” and “melt” such that they are more rigid or more fluid-like in their material properties, especially during development.^80^ As these tissues are structurally disordered, these fluid-to-solid transitions are immediately reminiscent of the glass transition. Furthermore, reformably bonded multicellular groups exhibit a variety of different signatures of the glass or jamming transitions,^198^ such as dynamic heterogeneities (when cellular rearrangements occur in a correlated, collective manner), caging (when cells are locked into their local neighborhood for long durations), heterogeneous intercellular force networks, and peaks in the vibrational density of states.^17,170,199^ However, unlike in a colloidal glass, which freezes when the density of particles is too high to continue the independent motion, internally generated forces (from, e.g., cell division, death, and growth) can cause tissue fluidization even at high cell densities.^171–173,186,200^ Interestingly, epithelial tissues can exhibit a unique density-independent form of the glass, as both fluid and solid states are confluent;^80^ recent studies suggest they undergo a rigidity percolation phase transition based on the number of nearest-neighbor cell contacts.^164^

### F. Intercellular Communication (Comparison to Nonreformable Intercellular Channels: Sec. II E)

Reformable bonds impact the types of intercellular channels that can be formed and used to pass nutrients between cells. This is because, unlike in the case of permanent bonds, cells generally do not connect their cytoplasms; as a result, they cannot form ultrastructural bridges, for example, with an endoplasmic reticulum that spans both cells. Instead, the types of junctions between cells are typically limited to ones that can be removed without causing cellular damage.

One prominent form of intercellular connection between reformably bonded cells is gap junctions or gap junction-like connections.^201^ These junctions are generally constructed as a pore in the cell membrane, where the rim of the pore is a complex protein structure that may selectively choose which types of ions and small molecules can pass between the two cells.^201^ When a pore on one cell aligns with a pore on a neighboring cell, they can dock together to straddle the gap between the two cells. These junctions tend to be small in diameter, on the order of a few nanometers, and are, therefore, only permeable for electrical currents and small molecules.^42,202^ Gap junctions are observed in all animals, reflecting their importance as a mechanism of intercellular communication.

The second common type of intercellular communication can occur in systems with any type of bond; cells may secrete small molecules and proteins into the surrounding environment. In this way, they may both read and write diffusible chemical gradients that may be cooperative or antagonistic.^150^ This indirect method of intercellular communication may allow multicellular groups to achieve a quorum,^134,203^ facilitate synchronized responses among many cells,^204^ and signal cellular differentiation.^205^

These two methods of cell-to-cell communication have benefits and drawbacks. On the one hand, public goods are susceptible to cheating individuals, while privately transferred goods are not. On the other hand, the number of cells an individual can interact with via secreted goods is effectively unlimited, while for direct methods it is explicitly limited by the number of cell contacts. The trade-offs of these two communication schemes, or their combination, are navigated by all multicellular lineages assembled with reformable bonds.

## IV. EVOLUTIONARY CONSEQUENCES OF INTERCELLULAR BOND TYPE

The first step in the transition to multicellularity is the formation of a multicellular group. Whether cell–cell bonds are permanent or reformable has a profound impact on the subsequent evolution of multicellularity. Broadly speaking, there appears to be a correlation between bond type and the evolution of “complex” multicellularity. In particular, large organisms with many cell types (used as a proxy for organismal complexity) have mostly evolved in multicellular organisms that made the transition to multicellularity with groups forming permanent cell–cell bonds (land plants, fungi, red algae, and brown algae).^8^ Animals may be a notable exception: they currently form complex multicellular groups with reformable bonds, but it is possible this is a derived trait, as little is known about the cellular topology of the transitional metazoans.

This pattern may arise from a number of ultimate evolutionary drivers. First, permanent cell–cell bonds result in largely clonal multicellular groups, which limits the potential for within-group social conflict^9,206,207^ and may favor the evolution of complex cellular interactions (i.e., those underlying multicellular development) resulting from exceptionally high across-generation partner fidelity. Second, recent work has shown how the network of interactions created by permanent cell–cell bonds can favor the evolution of cellular differentiation, by making it easier to direct resources to complementary specialists.^26^ Third, organisms that develop with permanent cell–cell bonds are more likely to be obligately multicellular, as opposed to those which spend most of their life cycle in a unicellular stage and aggregate in response to an environmental cue.^208^ All of these topics are well covered elsewhere in the literature. Instead of rereviewing this work, we will focus on a topic that has received comparatively little attention: understanding how the type of cellular bond affects the earliest steps in the transition to multicellularity. In particular, we discuss the role of different intercellular attachment mechanisms in the emergence of multicellular life cycles and heritable multicellular traits.

### A. Origin of Group-Level Reproduction

Life cycles provide a framework for understanding the origin of multicellularity.^209–212^ In particular, once groups of cells form, they must have a way of growing and reproducing if they are going to participate in a process of Darwinian evolution. How groups grow and reproduce can be formally described by a life cycle. There are a number of ways that extant multicellular organisms reproduce, but they can be broadly grouped into two classes: new groups either start with a single cell, or new groups start with multiple cells. In either case, multicellular reproduction depends on breaking intercellular bonds, enabling the two groups to separate. In the simplest cases, a multicellular group becomes two or more (smaller) multicellular groups. This can proceed via multicellular fission, fragmentation, or membrane rupture;^19,37,44,58,124,213,214^ in other cases, it can proceed via abscission or separation along a line between two previously defined groups of cells.^30^ It is important to note that some bond networks are more amenable to this kind of reproductive event than others. For instance, linear filaments and branched trees with nonreformable bonds achieve group-level reproduction whenever a single intercellular bond fractures. Permanently bonded neighbor networks cannot achieve reproduction so easily: other than the trivial case where an edge cell separates, multiple bonds must be broken to achieve group-level reproduction. However, groups with reformable bonds are even more physically constrained. Broken bonds can re-form, so if all of the necessary bonds do not break at the same time, two propagules can re-fuse together. Cell–cell strain arising from cellular reproduction is sufficient to drive multicellular reproduction in simple branched organisms with permanent bonds (e.g., snowflake yeast, which have a branched morphology^44^), allowing a life cycle to arise without any further evolutionary innovation or environmental input. By contrast, groups with reformable bonds, where the multicellular fracture is more difficult, generally reproduce either from the action of external physical forces (e.g., sloughing due to shear forces) or changes in the environment that trigger cellular dispersal, giving rise to the wholesale alternation between colony formation and cellular reproduction.^125,131,134,207,215^

### B. Origin of Multicellular Heritability

In order for selection acting on multicellular groups to drive multicellular adaptation, group-level traits must be at least somewhat heritable. While it has long been assumed that the origin of multicellular heritability requires a change in how genetic information is used,^216,217^ this outlook has recently been challenged.^76,218^ Instead, novel multicellular traits may emerge from changes in the traits of cells, and these emergent traits may themselves be remarkably heritable.

Clonal multicellular groups (which can arise with either permanent or reformable bonds but are more often found in permanently bonded groups) help facilitate the origin of novel, heritable multicellular traits. Mutations that change the attributes of individual cells (e.g., cell shape, metabolism, and age- or environment-dependent phenotypic responses) may have an emergent multicelluar phenotype once these cells are growing in a group—one that may not be at all functionally analogous to the cell-level trait itself.^76^ For example, mutations that increase the aspect ratio of snowflake yeast reduce the density of cellular packing in the cluster, which increases the size to which the group can grow before cell–cell fracture drives group-level reproduction.^16^ In clonal groups, these emergent multicellular traits covary with the causative cell-level mutation, allowing these emergent multicellular traits to be exceptionally heritable.^76^ To put it another way, when groups are clonal, emergent multicellular traits have a common genetic basis, which allows these traits to be recapitulated across generations and underpins their heritability. In fact, emergent multicellular traits may often be more heritable than their underlying cell-level analogues (despite the fact that the multicellular traits are epiphenomena and the cell-level traits are genetically encoded), due to the effects of averaging.^73,76^ That is, if the cell-level traits are somewhat noisy, the emergent multicellular trait can average over this noise and more precisely reflect the underlying genetic variation. While this logic holds for aggregative groups with reformable bonds as well, the heritability of emergent multicellular traits will be doubtless quite sensitive to within-group genetic diversity (though, to our knowledge, no work has directly examined this). Importantly, this emergent heritability can be maintained for long periods of directional selection. In the longest-running evolution experiment of nascent multicellularity, Bozdag *et al.*^25^ found that snowflake yeast clusters subject to 600 rounds of selection for larger size evolved to be ∼20 000 times larger than their ancestor, with gradual changes in cell-level traits (mainly cell length) underlying dramatically increased multicellular size and biophysical toughness.

### C. Noise, Topology, and Multicellular Robustness

What is the role of cellular spatial structure (i.e., geometric arrangements and topological intercellular connections) in ensuring that heritable multicellular traits are passed from parent to offspring? We have already highlighted how certain connection topologies can facilitate reproduction such that clonality is ensured. Yet, how do offspring generate functional multicellular properties anew? For example, even if multicellular groups are clonal, there are inherent fluctuations in cellular spatial structure due to the noisy process of multicellular growth. Do fluctuations in cell position, orientation, and/or connectivity destroy multicellular heritability?

It may be that fluctuations during the growth or assembly of multicellular groups counter-intuitively ensure that some structural properties are shared between parent and offspring, regardless of the intercellular adhesion mechanism. In particular, recent work has explored the role of random cellular assembly on the geometric arrangements of the cells.^18^ In experiments of permanently bonded snowflake yeast and *V. carteri*, and simulations of sticky aggregates, the distribution of cell neighborhood sizes followed precise maximum entropy predictions, so long as fluctuations were not too small. The cell-packing distribution is, therefore, a remarkably consistent multicellular property of multicellular groups, which arises without the need for developmental regulations and feedbacks. In principle, any nascent multicellular organism without developmental patterning will pack its cells according to this distribution. Furthermore, the consistency in cellular packing arising from maximum entropy considerations underlies the stability and predictability of emergent multicellular traits that rely on cell packing (i.e., group size upon which strain arising from cellular division results in fracture), providing a physical mechanism for their remarkable heritability.^76^

In a related vein, topological similarity can also propagate from parents to offspring without developmental patterning. In particular, unlike groups with reformable bonds, bond topology automatically propagates with permanent bonds. Upon bond fracture, the remaining bond network of the propagule is unchanged for groups with permanent bonds. Group topology is thus independent of the fragmentation process and only depends on the “rules” that govern the formation of nonreformable bonds. Conversely, the topology of groups with reformable bonds can be fundamentally changed by a fragmentation process, whereby many cellular rearrangements may occur. The topology of groups with reformable bonds thus depends both on the fragmentation process and subsequent rounds of reproduction. Each of these processes presents challenges and benefits. On the one hand, rearrangements can allow multicellular phenotypes to plastically adapt to their environment, while nonreformable bonds lock in an unchangeable topology. On the other hand, a particularly successful spatial structure can rapidly propagate through the combination of permanent intercellular bonds and bond fracture, especially in comparison to malleable reformable bonded structures.

## V. AMBIGUITIES IN THE REFORMABLE/NONREFORMABLE BINARY

One advantage of classifying bonds as either reformable or nonreformable is the clean distinction between these two classes. After breaking, a bond either can or cannot re-form. Nonetheless, in this section, we discuss some of the ambiguities that arise when sorting adhesion mechanisms with this classification scheme.

### A. Organisms That Have Both Reformable and Nonreformable Bonds

While all bonds are either reformable or nonreformable, multicellular groups are not constrained to only have one class of adhesion mechanism. For example, animals often initially develop with nonreformable bonds before switching to utilize reformable bonds for the vast majority of the developmental process. In some cases, animals maintain cytoplasmic bridges between somatic cells, a hallmark of permanent bonds;^47^ at the same time, these organisms have, for example, red blood cells that are not permanently bonded. In addition, while plant cells are generally permanently bonded, there are important cases where reformable bonds fuse two separate pieces together; examples include pollen attaching to stigma, the fusion of floral organs, and agricultural grafts.^30^ These are two of just many cases in which both reformable and permanent bonds exist. The distinction that we draw is thus to sort bonds, not organisms, into the classes of reformable and permanent.

Moreover, the initial class of intercellular bond may be distinctly important. In any given extant multicellular lineage, both reformable and permanent bonds may exist. However, it is unlikely that both types of bonds simultaneously evolve. Therefore, the initial evolution of groups is likely started with a single type of bond (either permanent or reformable), influencing the subsequent evolution of multicellularity. There may be merit in classifying multicellular lineages by the type of bond present at the transition to multicellularity, as opposed to only the types of bond displayed by extant representatives.

### B. Timescales

In some cases, reformable bonds that rarely, if ever, the break may behave similarly to nonreformable bonds. This may be especially true on short timescales, during which few, or no, bonds break. However, the important distinction is not if bonds break but if bonds can re-form after breaking. For example, adherens and tight junctions, during which animal cell membranes tightly fuse together, are common in epithelial tissues.^219^ Cells connected with these junctions may not easily separate, perhaps for their entire lives. Therefore, the structural contribution of adherens junctions to an organism may seem to capture properties that we would associate with permanent bonds more than reformable bonds. Nonetheless, such junctions are reformable: if the two cells disconnect, and then re-encounter one another, they can form a new junction. Alternatively, they could disconnect and form new junctions with other cells in the body. By contrast, permanent bonds cannot form without additional cell division or partitioning.

### C. Reformable Bonds That Connect Cytoplasms

In some cases, reformable bonds may be able to connect cell-to-cell cytoplasms, forming cytoplasmic bridges and pore structures that are reminiscent of permanent bonds. For example, some cells in mycelial networks can fuse together, connecting previously unconnected hyphal branches with fully functional septal channels, increasing the overall connectivity of the network.^81^ This style of bond formation is typical for wound healing processes in both fungi and plants.^30^ Moreover, in plants new bonds may be formed this way through intrusive growth, whereby one tissue layer grows into (and possibly through) another; one common example is pollen tubes, which grow into and fuse with stylar tissue.^220^ Because this class of bonds can re-form, in the sense that they can continue to fuse separate surfaces together, we classify these bonds into the reformable category. It is worth noting that reformable bonds that connect cytoplasms rely on cells locating one another, communicating, and homing; in fact, in mycelia there are spatially distinct regions of the network, which employ this method, and other regions that actively avoid crossing hyphae.^81^ Such behavior is a far more involved process than the permanent bonds, which typically connect two cells in a filament; it appears likely that the permanent bond formation mechanism evolved first, and then, the capability of “fusion” bonds evolved at a later adaptation.

## VI. PERSPECTIVE

It can be difficult to draw broad generalizations in biology, even more so when we are considering a major evolutionary transition that has taken many different paths, including lineages as diverse as animals, algae, fungi, and pack-hunting bacteria. Yet, all multicellular organisms share several things in common: they are composed of multiple cells, those cells are physically attached, and these groups of cells participate in a process of Darwinian evolution, gaining adaptations. In this study, we have shown how these types of cellular bonds can be generally grouped by whether they are reformable or nonreformable. While this difference is relatively simple, it has profound implications for the origin and evolution of multicellularity, constraining future evolutionary and biophysical dynamics. Sorting intercellular bonds into these two classes provides a framework by which we can begin to understand not just how multicellular organisms behave, but also how the simple act of forming cell–cell bonds affects their evolution. In particular, the class of adhesion mechanism impacts the earliest stages of nascent multicellularity, the emergence of the group as a Darwinian individual, and the long-term evolution of complex multicellular traits, such as cellular differentiation and communication.

Understanding how groups of cells become Darwinian entities is an active area of research.^18,25,44,76,124,207^ In particular, little is known about how nascent multicellular groups express heritable variation in multicellular traits in the absence of developmental genetics, which allows mutations to create novel and heritable multicellular traits. In this study, we show how permanent bonds provide one answer to this conundrum, conferring high levels of consistency in cellular-connection topology between parents and offspring. Therefore, groups with nonreformable bonds can possess emergent heritability of structural multicellular properties without requiring that these traits are constructed by a genetically regulated developmental process. Conversely, while groups with reformable bonds may not obtain these advantages, their topological malleability may provide advantages in fluctuating environments.^221,222^ Whether organisms evolve highly specialized multicellular structures, or remain diverse generalists, thus may be dependent on the mechanism of intercellular attachment.

Another multicellular trait that strongly depends on the attachment mechanism is group size. Fragmentation limits the size of groups; however, the number of bonds that must fracture for a group to break into two separate pieces strongly depends on the adhesion mechanism. For permanent bonds, group size is highly limited by fracture. In some cases, a single weakest link can fragment the entire organism. Conversely, reformable bonds can “heal,” limiting the impact of fragmentation on size. Instead, groups with reformable bonds are more prone to cheating cells, which may destroy the mechanical resilience of the group before it becomes large.^207,215^

Along with these nascent multicellular traits, the evolution of complex traits depends on the intercellular bond type; for example, to achieve sustainable large size, groups must evolve a means of importing nutrients and exporting toxins.^8^ The type of intercellular bond constrains the types of solutions that may emerge in response to these challenges. Groups with permanent bonds can readily form intercellular channels as connected cells already possess adjacent membranes. Once these channels are formed, cell–cell exchange of nutrients opens up the potential for specialization and division of labor, a class of behaviors that underlies many multicellular adaptations.^26,27,223^ Conversely, cells with reformable bonds generally form intercellular channels that are smaller and thus less effective for transport, using imperfect protein–protein interactions to preferentially attach to related cells. Groups with reformable bonds thus tend to trade less via robust intercellular channels. Instead, a common strategy for these groups is to use excreted goods in order to exchange nutrients. These goods are highly susceptible to cheaters, which make use of, but do not produce, common goods.^215^ On the other hand, common goods can also be readily exchanged with nonkin, forming complex metabolic networks.^224^ Thus, the biophysical constraints and opportunities presented by permanent and reformable bonds play important roles in the evolution of morphological and metabolic complexity. For all the reasons described above, it may be of little surprise that “complex” multicellular organisms (i.e., those with multiple cell types: plants, fungi, green algae, red algae, and brown algae) predominantly have permanent cell–cell bonds, while animals are the only lineage possessing complex multicellularity with (mainly) reformable bonds.

Another important reason that intercellular bonds may play multiple roles in the formation and maintenance of multicellular groups lies within the topic of mechanotransduction. In short, cells are known to sense the mechanics (like stiffness) of their surroundings and accordingly change their behavior.^225,226^ These surroundings include other cells that are within their vicinity. It seems likely that bond formation can both impact and be impacted by these cellular neighborhoods, leading to a complex coupling between cell behavior and multicellular assembly. The field of cellular mechanical sensing is, therefore, a growing and exciting front through which further biophysical understanding of initial multicellular evolution may be gained.

The evolution of multicellularity cannot be understood without considering its physics. Cells live in groups that are mechanically, topologically, geometrically, and functionally constrained by physical interactions, all of which are filtered and amplified by the lens of Darwinian evolution. This is a particularly promising time to work on this topic, as we have a rich assortment of natural experiments in multicellularity (>50 independently evolved lineages), and experimentally evolved and synthetically generated model systems.

## Data Availability

Data sharing is not applicable to this article as no new data were created or analyzed in this study.
